# Multifocal Choroiditis in a Diabetic Patient

**Published:** 2011-10

**Authors:** Alireza Hedayatfar, Touka Banaee

**Affiliations:** Noor Eye Hospital, Tehran, Iran; Assistant Professor, Mashhad University of Medical Sciences, Mashhad, Iran

## CASE PRESENTATION

A 48-year-old woman presented with floaters, photopsia, blurred vision and ocular pain in both eyes of a few days’ duration prior to referral. She had a history of diabetes mellitus for 10 years and was being treated with glibenclamide and metformin.

**Figure 1:**
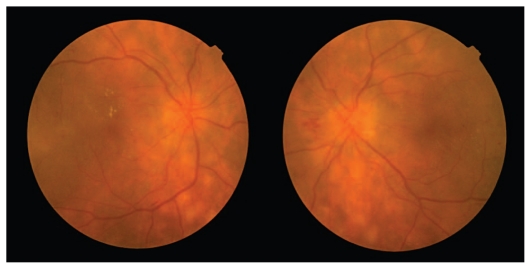
Fundus appearance at presentation.

*On examination, visual acuity was 20/160 and 20/50 in the right and left eyes, respectively with no significant refractive error. Both pupils were normally reactive and no afferent pupillary defect was present. Slit lamp examination disclosed 1+ cell in the anterior chamber, pigmentation of the anterior lens capsule, 2+ nucleus sclerosis and 1+ to 2+ vitreous cells in both eyes. Intraocular pressure was 16 mmHg in both eyes. Fundus examination revealed multiple well-circumscribed creamy-yellow subretinal lesions, mostly distributed in equatorial and peripapillary regions, together with dot and blot retinal hemorrhages, macular edema and florid neovascularization of the optic discs (*Fig. 1).

**Figure 2:**
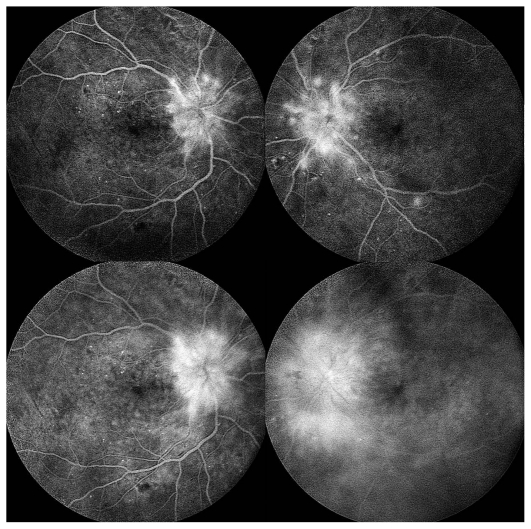
Fluorescein angiography at presentation (early and late frames).

**Figure 3:**
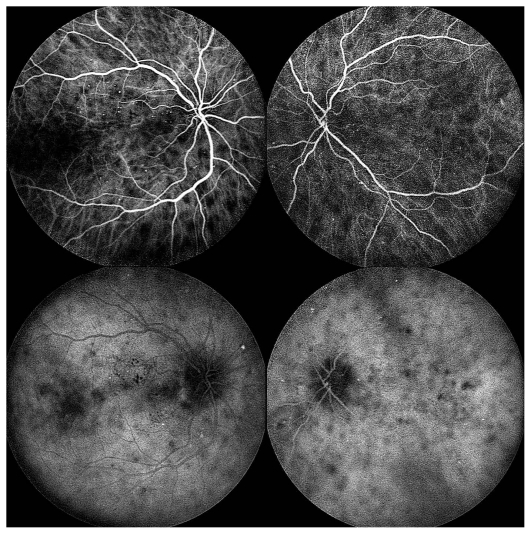
Indocyanine green angiography at presentation (early and late frames).

*Fluorescein angiography (FA) demonstrated early hypo- and late hyperfluorescence of the lesions (*Fig. 2). Apart from diffuse optic disc leakage, macular leakage was also notable. On indocyanine green (ICG) angiography, the lesions remained hypofluorescent through all phases of the angiogram (Fig. 3).

Laboratory investigations including VDRL, serum angiotensin-converting enzyme level and skin tuberculin test (STT), were within normal limits and HLA-A29 was negative. Chest X-ray was also unremarkable.

What are your differential diagnoses?

### Alireza Hedayatfar, MD

The presence of multiple creamy-white fundus lesions together with characteristic ICG angiographic features are in keeping with a diagnosis of “choroiditis”, which can broadly be categorized into choriocapillaropathies and stromal choroiditis. Multiple evanescent white dot syndrome (MEWDS), a primary inflammatory choriocapillaritis, has features in common with this case. It usually causes visual loss, photopsia and scotomata, and ICG angiography reveals numerous hypofluorescent spots mostly in the peripapillary region. However, MEWDS is usually unilateral and self-limited, which was not the case in this patient. Other primary inflammatory choriocapillaropathies such as acute posterior multifocal placoid pigment epitheliopathy (APMPPE) and serpiginous choroiditis, although bilateral in most cases, cause geographic or helicoidal subretinal lesions which are distinct from the well-circumscribed spot-like lesions seen in this patient. Vogt-Koyanagi-Harada (VKH) syndrome, a primary stromal choroiditis, has numerous hypofluorescent dark dots (HDD) on ICG angiography which resembles this case. However, the absence of neurologic/ auditory symptoms, serous retinal detachment, and stromal choroidal vasculitis (intermediate fuzzy stromal vessels and late diffuse choroidal hyperfluorescence on ICG) are against this diagnosis. Birdshot chorioretinopathy, another primary stromal choroiditis, may present similar to this case, but a negative HLA-A29 and lack of prominent retinal vasculitis make it less likely. The diffuse and rather regular distribution of the choroidal lesions (as depicted by HDD on ICG angiograms) and lack of laboratory evidence reduce the possibility of ocular tuberculosis (TB) and sarcoidosis in this case. This patient demonstrates combined features of choriocapillaropathies (choriocapillaris non-perfusion) and stromal choroiditis (HDD on ICG angiography) and may best be labelled as “multifocal choroiditis”.

### Touka Banaee, MD

The patient described herein is an interesting case of bilateral multifocal choroidal inflammation/infiltration combined with diabetic retinopathy. When uveitis especially, choroiditis occurs in a diabetic patient, there are several diagnostic and therapeutic challenges that must be addressed. One challenge is ruling out opportunistic infections of the choroid. The list of differential diagnoses for this patient may include the following.

1. Idiopathic inflammations of the choroid:
  - Birdshot chorioretinopathy
  - Multifocal choroiditis and panuveitis syndrome
2. Granulomatous inflammations and infections:
  - Sarcoidosis
  - Tuberculosis and atypical mycobacterial infections
  - Syphilis
3. Malignancies:
  - Intraocular lymphoma
  - Metastatic infiltration of the choroid
4. Fungal infections of the choroid:
  - Cryptococcus neoformans
  - Pneumocystis carinii
  - Coccidioides immitis
  - Sporothrix schenckii

Sarcoidosis, miliary tuberculosis and syphilis may lead to a picture similar to this case. However, normal systemic examination, negative laboratory tests and normal chest X-ray rule out all of these diagnoses. I myself would recheck the skin test with a double strength test when the clinical picture is compatible with tuberculous uveitis. This has not been performed for this case, but the clinical course (described below) proves the condition not to be tuberculosis because it would have aggravated under corticosteroid and immunomodulatory treatment.

In my opinion, intraocular lymphoma and choroidal metastasis are also unlikely due to the absence of neurological symptoms and the presence of significant vitreous inflammation. Furthermore, the clinical course makes such possibilities very unlikely; the patient did not develop systemic symptoms nor did the ocular condition aggravate during follow-up.

Atypical fungal infections such as those caused by *Cryptococcus neoformans*, *Pneumocystis carinii*, *Coccidioides immitis*, and *Sporothrix schenckii* may produce such a clinical picture, usually in the context of systemic involvement and immunosuppression. The normal systemic condition, normal chest X-ray, and the course of the disease make these diagnoses remote.

The most likely remaining diagnoses are the first category in the differential diagnosis listed above, namely idiopathic choroidal inflammations. Although a negative HLA-A29 makes the diagnosis of birdshot chorioretinopathy unlikely, the clinical picture of cream-colored, radially-oriented choroidal lesions with nearly same sizes in a middle-aged woman is compatible with a diagnosis of birdshot chorioretinopathy rather than multifocal choroiditis and panuveitis syndrome. Although HLA-A29 may be negative in 4 to 10% of cases with birdshot chorioretinopathy, certain findings are in favor of multifocal choroiditis and panuveitis syndrome. The hypofluorescent spots on ICG angiography in this case are smaller, of unequal sizes and more randomly distributed than what is seen in birdshot chorioretinopathy. Another clue is the development of late subretinal fibrosis as observed on fundus photographs and optical coherence tomography (OCT) images (depicted below). Multifocal choroiditis and panuveitis syndrome, and the idiopathic subretinal fibrosis syndrome are considered entities along one spectrum and treatment does not differ much between them.

**Figure 4:**
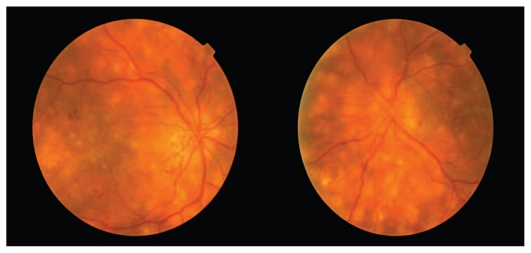
Fundus appearance 3.5 months after starting treatment.

*With a diagnosis of multifocal choroiditis and panuveitis, oral cyclosporine A 3 mg/kg per day and prednisolone 25 mg per day, along with topical betamethasone QID and tropicamide TDS were initiated in both eyes. Six weeks after starting treatment, the patient reported an improvement and visual acuity reached 20/120 in the right eye and 20/60 in the left one. Vitreous reaction and choroiditis diminished but neovascularization of the discs remained unchanged. At this time, prednisolone was gradually tapered to 12.5 mg and then to 7.5 mg per day and cyclosporine was continued at 200 mg daily. Two months later, the patient returned with deterioration of vision in both eyes to 20/200. Despite control of the inflammation in both eyes, disc neovascularization persisted (*Fig. 4), therefore full scatter panretinal photocoagulation (PRP) was performed.

Do you agree with performing PRP at this stage?

### Alireza Hedayatfar, MD

The main area of inflammation in multifocal choroiditis is the choroid. Development of retinal lesions and vitreous infiltration is a secondary process, therefore a quiet anterior chamber and vitreous does not necessarily indicate well-controlled inflammation. ICG would be a more precise and reliable measure to evaluate disease activity. In fact, visual deterioration and progression of fundus lesions (apparent by comparison of the two successive color fundus photographs) are indicators of insufficient control of inflammation. Nodularity and irregular thickening of the retinal pigment epithelium (RPE), as revealed on OCT images, further support the presence of an active state. In a burnt out state, the corresponding scars would have caused atrophic changes.

Persistent disc neovascularization may be attributed to both retinal ischemia due to diabetic retinopathy and/or persistent ocular inflammation. The etiopathology of neovascularization in inflammatory diseases (secondary to inflammatory cytokines) is different from diabetic retinopathy (subsequent to retinal ischemia); therefore, further suppression of inflammation would have been indicated before attempting laser photocoagulation which in turn, may exacerbate macular leakage. Personally, I think PRP would have been indicated only if an ICG angiogram had already revealed an inactive state.

### Touka Banaee, MD

I completely agree with starting a high dose of steroids with low dose cyclosporine, and tapering steroids a few weeks later. In a diabetic patient, one must bear in mind the possibility of enhanced drug complications and monitor the patient closely. Fortunately, the authors have handled the case smoothly and without complications.

Neovascularization of the optic disc can be due to diabetes or uveitis. There are not many dot and blot hemorrhages or other signs of diabetic retinopathy in the fundus photographs, and the neovascularization may be assumed to be secondary to uveitis. If disc neovascularization is due to uveitis, then it may regress upon control of the inflammation. If it does not regress after controlling disease activity, PRP is indicated as had been done in this patient.

**Figure 5:**
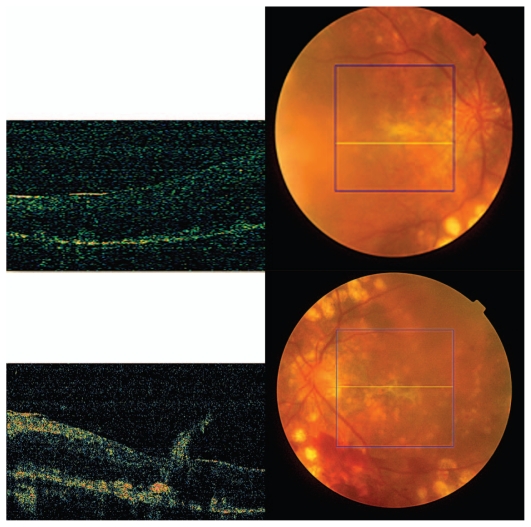
OCT and fundus photography 8 months after starting treatment.

**Figure 6:**
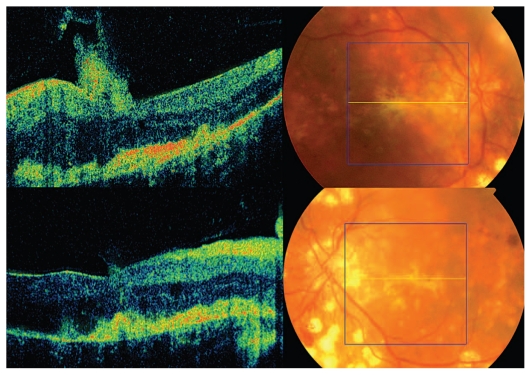
OCT and fundus photography 26 months after starting treatment.

*During the next 4 months, the patient’s vision deteriorated progressively and visual acuity dropped to 20/400 in both eyes despite control of the inflammation. At this time mild vitreous and pre-retinal hemorrhages were detected in her left eye. OCT showed increased central macular thickness in both eyes (*Fig. 5). At this stage, an intravitreal injection of bevacizumab was performed in the left eye; 18 months later, visual acuity decreased to 5/200 in the right eye and 20/400 in the left eye. On fundus examination pre-retinal and fresh vitreous hemorrhages were visible in her left eye (Fig. 6).

*Vitreoretinal* **s***urgery was performed on the left eye. However, due to a cataract and permanent macular damage, visual acuity did not improve significantly. The patient eventually underwent phacoemulsification with posterior chamber intraocular lens implantation in both eyes. At final examination, visual acuity improved to 20/200 with complete control of the inflammation while she was on medical therapy with cyclosporine 75 mg per day and prednisolone 7.5 mg every other day.*

What would you suggest for further management?

### Alireza Hedayatfar, MD

I would look for signs of subclinical disease activity; combined FA/ICG angiography would be a good option at this stage. If the disease is not fully controlled and HDDs are still present on ICG angiography, one could increase the dose of cyclosporine A, switch to another immunosuppressive agent (e.g. azathioprine) or add one to the current regimen. If there is no disease activity, the patient can be kept on the current medications. Lesions that remain hypofluorescent throughout the intermediate and late phases of angiograms may correspond to areas of choroidal scarring and should not be interpreted as indicators of disease activity. Evidence for early choroidal neovascular membrane (CNVM) should also be sought for. Meanwhile, residual optic disc neovascularization could be managed by further laser photocoagulation and/or intravitreal anti-vascular endothelial growth factor (VEGF) injections, once the inflammation is quiescent. Anti-VEGF agents offer the advantage of simultaneous treatment of an associated CNVM.

### Touka Banaee, MD

The continued decrease of vision in this case during follow up has not been adequately addressed. This may be due to macular ischemia secondary to diabetic retinopathy, which we do not have a fluorescein angiogram to confirm. No thickness maps of the macula are presented, and OCT images show some degree of macular edema which seems unlikely to lead to a significant decrease in vision. There is subretinal tissue just under the fovea in both eyes, which may be due to fibrosis or formation of CNVM. There are no accompanying fluorescein angiograms to reveal the cause but this sub-foveal tissue can be considered as the main cause of visual loss. Some epiretinal tissue with vitreomacular traction is also present in both eyes.

It has been stated that disease activity was controlled, an impression which seems to be based on clinical findings. It would be prudent to perform FA and ICG angiography to exclude the presence of subclinical choroidal inflammation. Furthermore, these studies would help delineate the cause of visual loss; whether there had been progression of macular ischemia, or formation of CNVM.

The left eye received an injection of bevacizumab. There are no notes to the indication for injection. If it had been done to treat macular edema, it is not clear what the result has been and why it was not repeated. Such injections are sometimes performed to induce regression of active neovascularization as a temporary measure before PRP, but this seems unlikely since PRP was performed several months later.

The authors have not stated the indication for vitreoretinal surgery. The vitreous hemorrhage had been mild and the uveitis had been under control. I suppose that, it surgery was done to relieve vitreomacular traction and macular edema. Performing a fluorescein angiography before surgery would have helped prognosticate the visual results by assessing the state of the perifoveal capillary net, and presence or absence of subretinal neovascularization and leakage.

